# OA30 Distinguishing active tuberculosis from flare in a child with severe systemic lupus erythematosus: Diagnostic and therapeutic challenges in a high TB-burden setting

**DOI:** 10.1093/rap/rkag113.030

**Published:** 2026-11-03

**Authors:** Atreya Ramanan, Pavneet Kaur, D R Rudra, Manisha Jana, Narendra Bagri

**Affiliations:** Newcastle University, Newcastle, United Kingdom; All India Institute Of Medical Sciences Delhi, New Delhi, India; All India Institute Of Medical Sciences Delhi, New Delhi, India; All India Institute Of Medical Sciences Delhi, New Delhi, India; All India Institute Of Medical Sciences Delhi, New Delhi, India

## Abstract

**Introduction:**

Patients with systemic lupus erythematosus (SLE) are at increased risk of infections due to disease-related immune dysfunction and immunosuppressive therapy. In the absence of microbiological evidence, distinguishing chronic infections such as tuberculosis (TB) from a lupus flare can be a diagnostic challenge, particularly in high TB-burden countries like India.

**Case description:**

A 15-year-old girl being managed for SLE for the past 4 years, presented with persistent fever for three months, during the course of treatment (on hydroxychloroquine, tapering steroids, and mycophenolate mofetil). She was evaluated for disease activity and chronic infections including TB, considering the high burden setting. There were no fresh clinical concerns apart from fever. However, she was found to have a positive tuberculin skin test, with cervical and necrotic hilar lymphadenopathy (Fig 1).

Nucleic acid-based tests failed to detect tuberculosis in the sputum, gastric aspirate, and lymph node samples. Nonetheless she was started on anti-tubercular therapy (ATT) considering clinicoradiological tuberculosis. ATT had to be modified for transaminitis a month into treatment.

Four months later, the patient developed fever recurrence, new-onset vasculitic cutaneous lesions (Fig.2), arthritis, anxiety neurosis, and nephrotic range proteinuria with class V lupus nephritis on renal biopsy. The SLEDAI score was computed to be 34, meriting intensive immunosuppression despite the concomitant setting of infection. There was no worsening of the radiological findings of tuberculosis. Thus, the possibility of immune reconstitution or drug-resistant tuberculosis seemed less likely.

Management: The modified anti-tuberculous therapy (ATT) was continued and the flare was treated with intravenous methylprednisolone pulse (30mg/kg/dose) for 3 days, followed by intravenous cyclophosphamide (500mg/m2). The clinical and serological activity markers, including transaminitis, improved thereafter.

**Discussion:**

**Discussion:** This case highlights the diagnostic complexity of diagnosing TB in patients with SLE, where clinical features due to infection, disease, and immunosuppression may be difficult to discern from each other. A similar conundrum is observed in therapy, where diagnostic delay leads to therapeutic dilemma. Careful multidisciplinary discussion was crucial to balance the risks of uncontrolled lupus activity against progression of active tuberculosis. In such cases, cautious immunomodulation is warranted, with parallel treatment of concomitant infections.

**Key learning points:**

• Active tuberculosis should remain a key differential diagnosis in patients with SLE presenting with constitutional symptoms, even when initial molecular investigations are negative.

• Escalation of immunosuppression should be guided by objective evidence of lupus activity and multidisciplinary assessment when infection cannot be confidently excluded.

• Successful management may require parallel treatment of active infection and severe autoimmune disease, balancing the risks of uncontrolled inflammation against progression of infection, in order to achieve optimal patient outcomes.

